# Mechanical properties of sustainable concrete comprising various wastes

**DOI:** 10.1038/s41598-023-40392-2

**Published:** 2023-08-14

**Authors:** Ramy M. Reda, Hoda S. E. Mahmoud, Seleem S. E. Ahmad, Hossam El-Din M. Sallam

**Affiliations:** 1 Civil Engineering Department, Higher Technological Institute (HTI), 10th of Ramadan City, 44629 Egypt; 2 Materials Engineering Department, Zagazig University, Zagazig, 44519 Egypt

**Keywords:** Engineering, Civil engineering

## Abstract

Due to the rapid increase of pollution around the world, the disposal of waste materials such as granite powder (GP), iron powder (IP), brick powder (BP), and waste plastic particles (PP) is a major environmental problem in the entire world. Utilizing these industrial waste materials has many advantages for the construction industry regarding cost-effectiveness and the sustainability of natural resources. This investigation examined the addition of GP, IP, BP, and PP as a fine aggregate with ratios of 5%, 10%, 15%, and 20% of sand in producing and assessing sustainable concrete. The static properties, i.e., compressive, tensile, flexural strength, and dynamic properties using the drop-weight impact test, were evaluated of such materials. The results showed that using IP as a partial replacement enhances both static and dynamic properties of concrete; the enhancement kept increasing up to 20% of IP, and the compressive, tensile, flexural strength, and impact energy increased by 8.4%, 12.5%, 8.5, and 125%, respectively. Therefore, IP can be suggested to replace sand by up to 20%. Using PP up to 15% enhanced the impact energy at failure by about 225%. It also observed that the optimum value for GP and BP was 10%. When using 10% GP the increase in the compressive, tensile, flexural strength, and impact energy was 11.7%, 25%, 21.5%, and 100%, respectively, while it increased by 12.9%, 7.6%, 15.4%, and 63% respectively when using BP.

## Introduction

Sustainability is achieving our needs without consuming non-renewable resources. Since construction is one of the largest fields consuming limited resources, sustainability has recently accelerated due to non-renewable resource constraints^1–4^. On the hand, the massive use of the natural material is diminished, and the cost increases over time^5–8^. Several studies have emphasized the problem of the high consumption of natural materials and recommended using various wastes to produce sustainable construction materials^7–22^.

Miah et al.^23^ examined the effect of replacing sand with iron powder (IP) partially on the mortar's mechanical properties; the replacement ratios were 0, 5, 10, 15, 20, 30, and 50%. This study concluded that the optimum value of IP was 30%. Noori et al.^24^ investigated the influence of replacing 6%, 12%, 18%, 24%, and 30% of the fine aggregate with IP on the concrete strength. The results showed that the concrete strength increased with the replacement ratio up to 12%. Dhanabal et al.^25^ investigated the influence of replacing 30% of sand with iron ore tailing constant for all mixes and 0%, 10%, 20%, and 30% of cement with glass powder (GP) on the strength and durability of concrete. The research suggested using 10% GP and 30% iron ore tailing, which enhances the concrete strength. On the other hand, water absorption and workability decreased with the increase in GP^25^. Rao et al.^26^ investigated the influence of replacing 20% of sand with ground granular blast furnace slag and cement with 6% nano-silica and 10% rice husk ash on the strength and durability of concrete. Ghannam et al.^27^ replace 5%, 10%, 15%, and 20% of sand with granite powder (GP) and IP. They concluded that 10% was the optimum value of GP. On the other hand, using 20% of IP was the best ratio for increasing the concrete strength. The same conclusion was made by Abraham^28^. Mhamal et al.^29^ studied the effect of using marble powder and GP to partially replace fine aggregates in concrete. A mixture of marble and granite in the ratio of 0.5:0.5 was used to replace sand with different percentages; 10%, 20%, 30%, 40%, and 50% by weight. The results showed that a 20% replacement ratio of fine aggregate with marble and granite increased the compressive strength by 32% compared to conventional concrete; considering the cost of sand, it saved about 20–40% of the sand cost. Kala^30^ replaced the sand with 25% of GP in concrete. Salvi et al.^31^ replaced 0%, 5%, 10%, 20%, and 30% of fine aggregate with GP to study its effect on compressive strength. The test results showed that compressive strength increased up to 10% of GP; after that, compressive strength decreased with increasing GP%. The research stated that the replacement of granite powder reduces the usage of sand and enhances natural resources. Prakash et al.^32^ used recycled plastic particles (PP) to partially replace fine aggregates with ratios (0%-20%) by weight to study concrete's strength. The test results showed that the strength decreased with increasing waste PP. Waste PP showed better resistance to abrasion when compared to the control mix; it showed that the addition of waste plastic didn’t affect the workability; from the carbonation test, it was observed that the depth of carbonation when used (2.5–12.5%) of PP was lower than or equivalent in case of water cement ratio 0.4 and 0.3, on the other hand, the least amount of carbonation obtained when using 7.5% to 10% of PP. Amaluet al.^33^ used 10–20% PP in concrete. The concrete strength decreased with the increase of PP. PP increases workability because plastic has lower absorption of water in concrete^33^. Shyam et al.^34^ studied the benefit of replacing 5%, 10%, 15%, and 20% of sand with waste plastic (High-Density Polyethylene powder) on compressive, split tensile, or flexural strength, either non-destructive test of hardened concrete, based on the results compressive, flexural, and split tensile strength decrease with the addition of waste plastic after 5%. Replacement of 5% waste plastic increased the compressive, flexural, and split tensile strength of 16.6%, 46.34%, and 22.8%, respectively; the workability decreased with increases in waste plastic^34^.

Since there is a variance in the replacement ratios used by researchers and disagreement in the optimum ratios, it was necessary to study the most used ratios and confirm their results. On the other hand, since the dynamic properties have not been studied extensively, it was necessary to cover this part with different wastes. Therefore, and complementary to previous studies, this research aims to carry out a scientific search on using waste materials in concrete as a step towards sustainability in construction. The researchers studied the effect of using GP, IP, brick powder (BP), and waste PP as a partial replacement of sand with ratios 5%, 10%, 15%, and 20% on the concrete strength under static and dynamic loadings. Furthermore, the novelty of this work is studying the compressive strength of mortar with the complete replacement of sand (100%) by these wastes.

## Experimental procedures

### Materials

**Figure 1:**
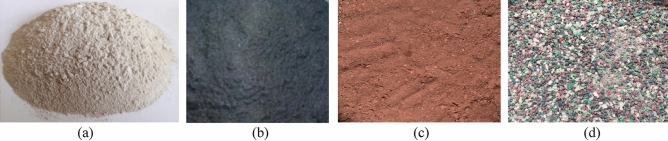
Replacement materials: (**a**) GP, (**b**) IP, (**c**) BP, and (**d**) PP.

**Figure 2:**
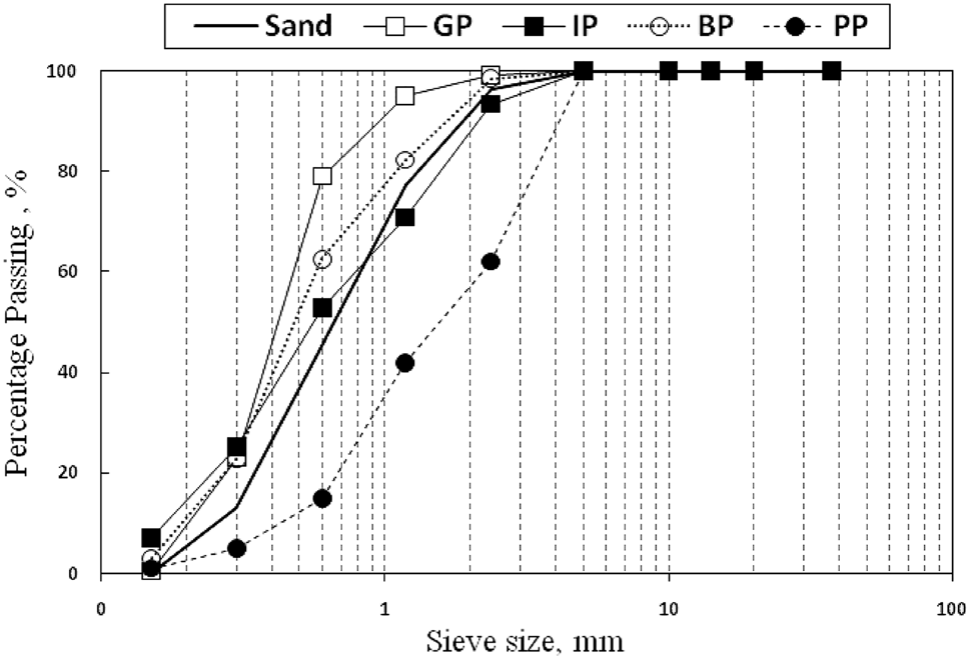
Sieve analysis of sand, GP, IP, BP, and PP.

Type I Ordinary Portland cement grade 52.5N was used according to ASTM C494. Dolomite with a maximum nominal size of 20 mm was used as a coarse aggregate, the specific gravity of the dolomite was 2.61, and the fineness modulus was 4.32. Natural local siliceous sand with a specific gravity of 2.55 and fineness modulus of 2.73 was used. Waste materials; GP provided from the crushing and polishing of granite, IP provided by steel industries, BP supplied from the manufacturing of bricks, and waste PP of Polyethylene terephthalate, as shown in Fig. 1, were used as a partial replacement of sand by volume with ratios 5%, 10%, 15%, and 20%. Physical properties such as specific gravity and fineness modulus of the sand, granite powder, iron powder, brick powder, and waste plastic particles are shown in Table 1. The sieve analysis for sand and the other waste materials is shown in Fig. 2. The chemical compositions of GP, IP, BP, and sand were listed in Table 2 as measured in the National Research Center labs, Dokki, Cairo, Egypt.

**Table 1:** Physical properties of the sand and different replacement materials.

Properties	Specific gravity	Fineness modulus
Sand	2.55	2.73
GP	2.55	2.46
IP	3.5	2.3
BP	2.5	2.3
PP	1.02	4.45

**Table 2:** Chemical composition of replacement materials.

Chemical compound %	SiO_2_	TiO_2_	Al_2_O_3_	Fe_2_O_3_	MnO	MgO	CaO	Na_2_O	K_2_O	P_2_O_5_	Cl	SO_3_	L.O.I
GP	70.5	0.47	13.47	3.58	0.07	< 0.01	1.10	3.82	4.06	0.06	< 0.01	< 0.01	2.52
IP	4.20	0.62	2.68	88.5	2.21	0.2	0.40	0.67	1.68	0.43	< 0.01	4.5	1.01
BP	63.24	0.94	13.82	7.03	0.126	2.09	3.62	1.43	0.89	0.35	0.21	2.88	2.62
Sand	80.96	–	11.64	1.84	–	0.52	3.45	–	1.41	–	–	–	–

### Preparation of test specimens

**Figure 3:**
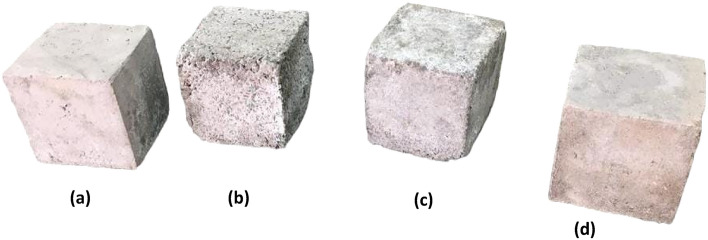
Mortar cubes: (**a**) GP cube, (**b**) PP cube, (**c**) IP cube, and (**d**) BP cube.

**Figure 4:**
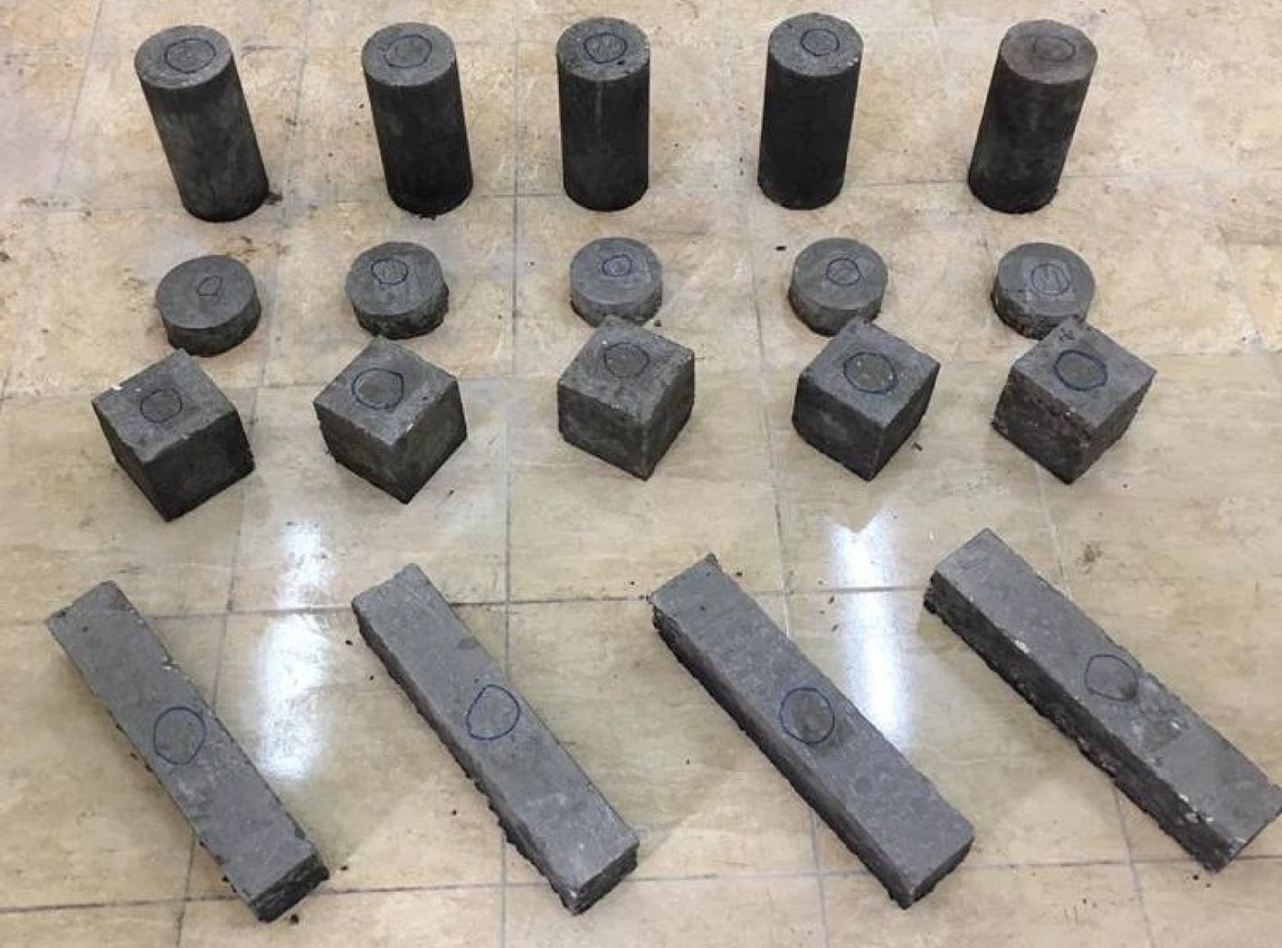
Concrete specimens for compressive, tensile, flexural, and impact strength.

Two sizes of cubic specimens were cast and tested to determine the compressive strength of both mortar and concrete. Twenty-five cubes of dimension 75 × 75 × 75 mm using different fine aggregates, i.e., sand and complete replacement of waste materials, GP, IP, BP, and PP, as shown in Fig. 3, were cast to determine the compressive strength of mortar after 28 days. In the case of concrete, a total of eighty-five cubes of dimension 150 × 150 × 150 mm were cast with a partial replacement of sand with GP, IP, BP, and PP by ratios 0%, 5%, 10%, 15%, and 20% and tested after 28 days. Eighty-five 150 mm diameter and 300 mm height cylinders were cast to determine the indirect tensile strength. To determine flexural strength, eighty-five 10 × 10 × 500 mm beams were cast and tested under flexural load. All specimens are tested using a universal testing machine of 1000 kN maximum capacity calibrated annually. Finally, eighty-five discs 150 mm in diameter and 60 mm in height were cast and tested under impact load according to ACI Committee 544 to determine the impact strength using calibrated drop-weight impact apparatus, as shown in Fig. 4. After cast, the specimens were left in the molds for 24 h, then removed from the molds and cured in a water tank for 28 days. The identifications of these mixes were as follows: (M-X) where M: replacement materials type (GP, IP, BP, and PP) and X: refers to replacement materials ratio (0%, 5%, 10%,15%, and 20%),

### Mix proportion

The mix proportions of the mortar with sand and different alternative materials are listed in Table 3, and the water-cement ratio was 0.40, and the target compressive strength equals 25 MPa. In the case of concrete, five concrete mixes with a fixed water-cement ratio equal to 0.50 and target compressive strength equal to 30 MPa for the control mix, the different mixes were made by varying the replacement ratio of the sand by the replacement materials with ratios 0%, 5%, 10%, 15%, and 20% by volume; the mix proportions of the five concrete mixes were listed in Table 4.

**Table 3:** Mix proportion of one cube of mortar (gm).

MIX	Cement (gm)	Water (gm)	Fine aggregate (gm)				
			Sand	GP	IP	BP	PP
M1	185	74	555	–	–	–	–
M2	185	74	–	555	–	–	–
M3	185	74	–	–	762	–	–
M4	185	74	–	–	–	544	–
M5	185	74	–	–	–	–	222

**Table 4:** Mix proportion for concrete (kg/m^3^).

MIX	Cement (kg)	Water (Lit)	Coarse aggregate (kg)	Fine aggregate (kg)				
				sand	GP	IP	BP	PP
S-0	350	175	1200	600	–	–	–	–
GP-5	350	175	1200	570	30	–	–	–
GP-10	350	175	1200	540	60	–	–	–
GP-15	350	175	1200	510	90	–	–	–
GP-20	350	175	1200	480	120	–	–	–
IP-5	350	175	1200	570	–	41.18	–	–
IP-10	350	175	1200	540	–	82.36	–	–
IP-15	350	175	1200	510	–	123.54	–	–
IP-20	350	175	1200	480	–	164.72	–	–
BP-5	350	175	1200	570	–	–	29.41	–
BP-10	350	175	1200	540	–	–	58.82	–
BP-15	350	175	1200	510	–	–	88.23	–
BP-20	350	175	1200	480	–	–	117.64	–
PP-5	350	175	1200	570	–	–	–	12
PP-10	350	175	1200	540	–	–	–	24
PP-15	350	175	1200	510	–	–	–	36
PP-20	350	175	1200	480	–	–	–	48

S = sand, GP = granite powder, IP = iron powder, BP = brick powder, and PP = plastic particles.

### Ethical approval

This article does not contain any studies with human participants or animals performed by any of the authors.

## Results and discussion

### Compressive strength

#### Compressive strength of mortar

**Figure 5:**
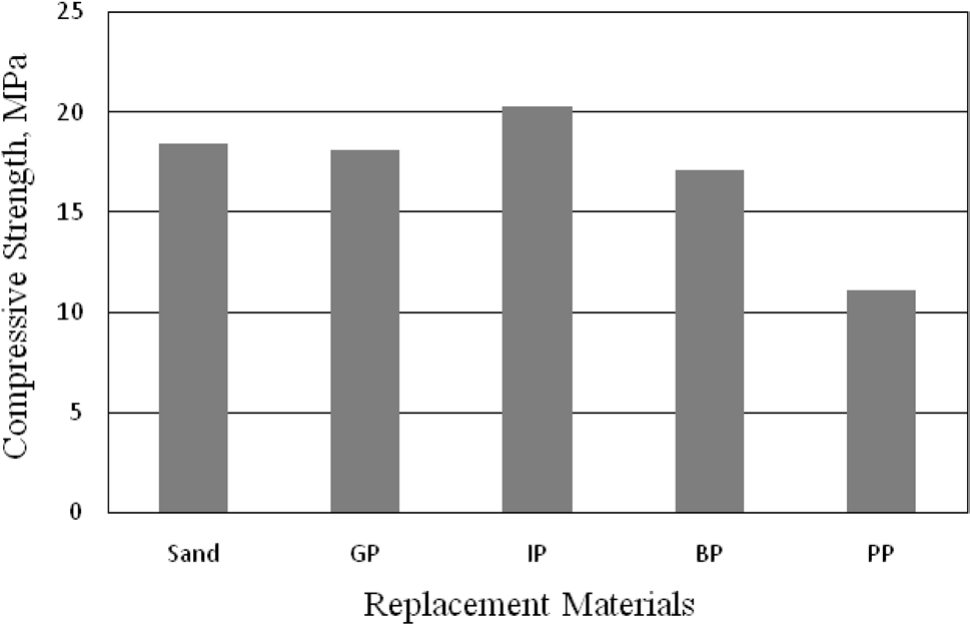
Compressive strength of mortar.

The influence of total sand replacement by GP, IP, BP, and PP on the compressive strength of mortars after 28 days is listed in Table 5 and shown in Fig. 5; the compressive strength was compared with the reference mortar (mortar with sand as a fine aggregate).

**Table 5:** Compressive strength of mortar.

	Replacement type				
	Sand	GP	IP	BP	PP
Compressive strength (MPa)	18.4	18.1	20.3	17.1	11.1

**Figure 6:**
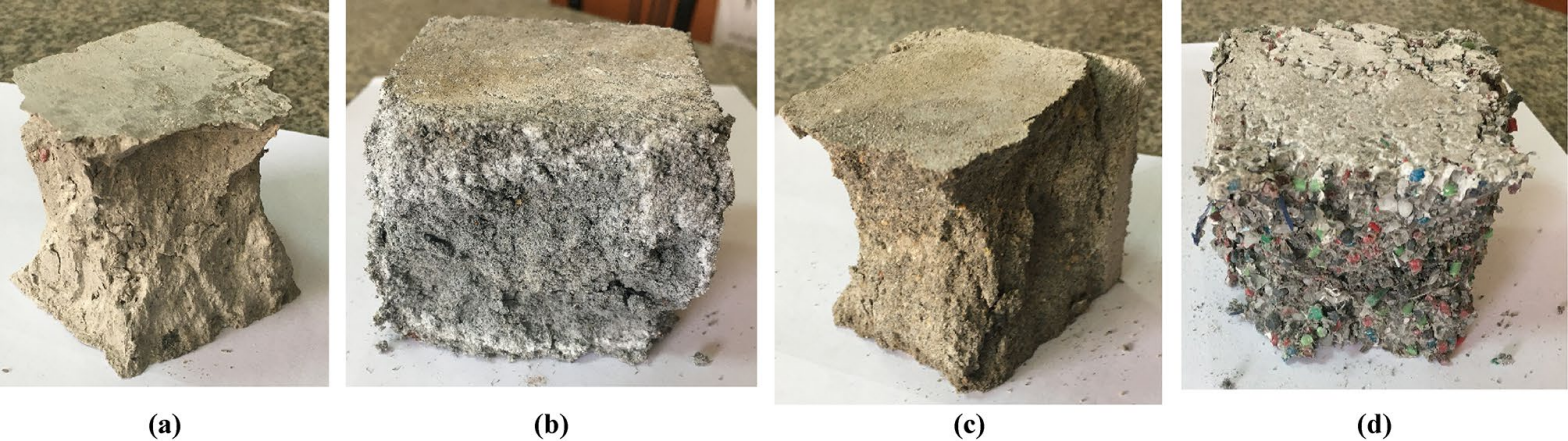
Failure shape of mortar cubes: (**a**) GP cube, (**b**) IP cube, (**c**) BP cube, and (**d**) PP cube.

The compressive strength of sand mortar was 18.4 MPa; replacing the sand with GP gives nearly the same results as sand mortar, as shown in Fig. 5. However, replacing the sand with IP increased the compressive strength of mortar, the compressive strength of IP mortar was increased by 10.3%. The increase in compressive strength may be due to the higher strength, rough surface, and angular shape of IP particles compared to sand particles, which leads to obtaining a strong interfacial transition zone between the fine aggregate and the cement paste, then improving the strength^23^. On the other hand, replacing the sand with BP and PP decreased the compressive strength of mortar by 7.1% and 39.7%, respectively see Fig. 5. The significant decrease in compressive strength for PP may be due to smooth and regular surface, a weak bond between the PP plastic particles and mortar leading to reduce the compressive strength of concrete. The same result was obtained by Kumar and Kumar^35^. They stated that plastic has poor bonding properties or low adhesion, leading to a weak bond between PP and cement and decreasing compressive strength^35^. The same trend of results obtained in the compressive strength of concrete, as mentioned in the next section, the shape of failure of the mortar cubes were shown in Fig. 6.

#### Compressive strength of concrete

**Figure 7:**
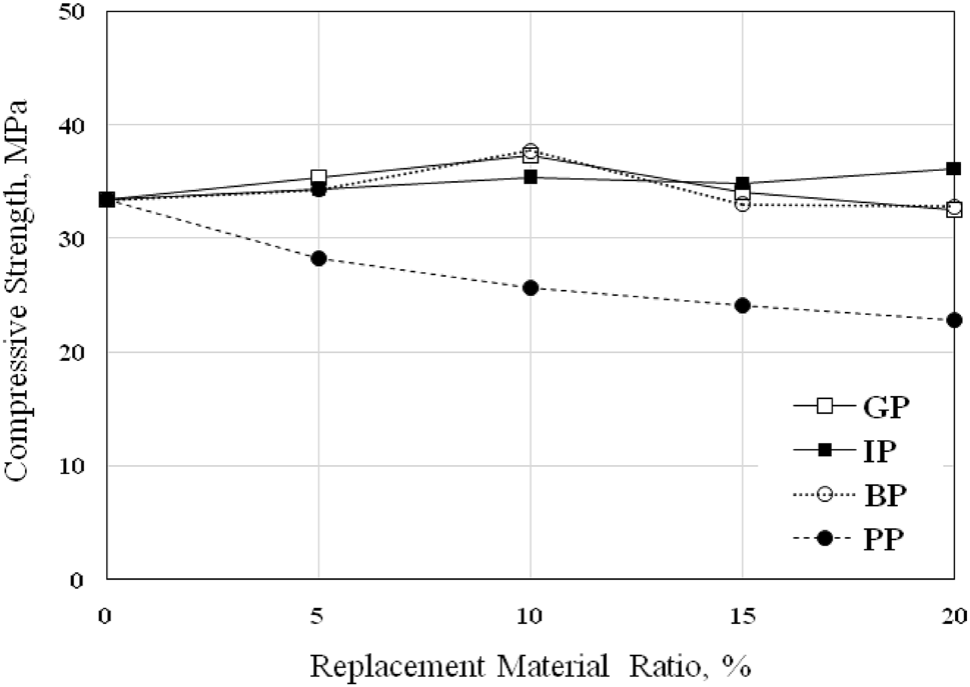
Compressive strength of concrete *f*_*c*_ with various replacement ratios.

The compressive strength, *f*_*c*_, of the cubic specimens was determined after 28 days of curing; the *f*_*c*_ results for control specimens and specimens with different percentages of GP, IP, BP, and PP were listed in Table 6 and shown in Fig. 7.

**Table 6:** Compressive strength of concrete, *f*_*c*_*.*

MIX	Replacement Ratio (%)	Compressive Strength,* f*_*c*_*,*(MPa)				
		0	5	10	15	20
GP		33.4	35.3	37.3	34.1	32.5
IP		33.4	34.3	35.3	34.85	36.2
BP		33.4	34.3	37.7	33.0	32.8
PP		33.4	28.2	25.6	24.1	22.8

As seen in the figure, increasing GP and BP ratios increase the *f*_*c*_ values of concrete when using 5% and 10% ratios; the same results were observed by^27–31^. The increase in strength ranged from 5.7% for GP-5 to 11.7% for GP-10 and increased from 2.7% to 12.9% for BP-5 and BP-10, respectively. The increase of the *f*_*c*_ to 10% might cause by the small particles size of GP and BP compared with sand particles, as seen in Fig. 2, which leads to fewer voids and a higher density of concrete; the GP and BP will fill the void between aggregate, in addition to the surface area of GP and BP particles will be larger than sand which will lead to increase the compressive strength due to the increase of the bond area. After that, the compressive strength decreased by increasing the replacement ratios. The strength decreased by 2.1% and 2.7% for GP-15 and GP-20, respectively, and decreased by 1.2% for BP-15 and 1.8% for BP-20. Increasing the replacement ratio of GP and BP above 10% made the surface area larger than that concrete can cover. As discussed, the BP increased the compressive strength when used with ratios 5% and 10% and gives so closed *f*_*c*_ to control mix when using 15% and 20% ratios; this means that the optimum percentage of GP and BP to achieve the maximum increase in *f*_*c*_ was 10%. Close to these results, using 15% of GP in concrete has a small enhancement effect, and 20% of GP gives an ineffective reduction in the *f*_*c*_.

Vijayalakshmi^15^ found that 10% GP is an optimum ratio to use in concrete; the decrease in compressive strength after 10% because of the increasing specific area and angular texture of granite powder, which leads to a noticeable loss in workability of the concrete resulting poor compaction, so increases in porosity which led to the decreasing in compressive strength^15^. Siva et al.^36^ observed that using 10% GP increased compressive strength by 40% compared to conventional concrete and using 20% and 30% GP increased compressive strength by 24% and 4%, respectively, compared to conventional concrete.

On the other hand, using IP as a sand replacement increased the concrete's compressive strength; the strength increased by 3%, 5.7%, 4.3%, and 8.4% for IP-5, IP-10, IP-15, and IP-20, respectively. Using IP gives an increasing effect at all ratios; the same observation was obtained in mortar. Full replacement of sand by IP enhances the compressive strength of mortar which increase by 10.3%; this means that the optimum percentage of IP was 20% to achieve the maximum enhancement in concrete; the same observation was obtained by Ghannam^27^. He^27^ found that the compressive strength increased by 13.1%, 19.0%, 32.7%, and 33.2% when using IP with ratios of 5%, 10%, 15%, and 20%, respectively. He stated that the increase in compressive strength may be caused by the surface geometry, the gradation, and the higher percentages of iron oxide^27^. However, Miah et al.^23^ found that replacing 5%, 10%, 15%, 20%, 30%, and 50% of IP increases the compressive strengths of mortar at 28 days by 12.8%, 25.8%, 26.7%, 29%, 29%, and 9.3%., respectively.

On the contrary, using PP as a replacement of sand decreased the *f*_*c*_ in all ratios; the *f*_*c*_ decreased by 15.6%, 23.4%, 27.8%, and 31.7% for PP-5, PP-10, PP-15, and PP-20, respectively. The same trend was obtained in the *f*_*c*_ of mortar which decreased by 39.7% when the sand was fully replacement by PP; this means that the PP is not effective to be used in concrete because of poor bonding properties between PP particles and mortar due to the smooth and regular surface, the same results were obtained by^32–34^.

### Splitting tensile strength

**Figure 8:**
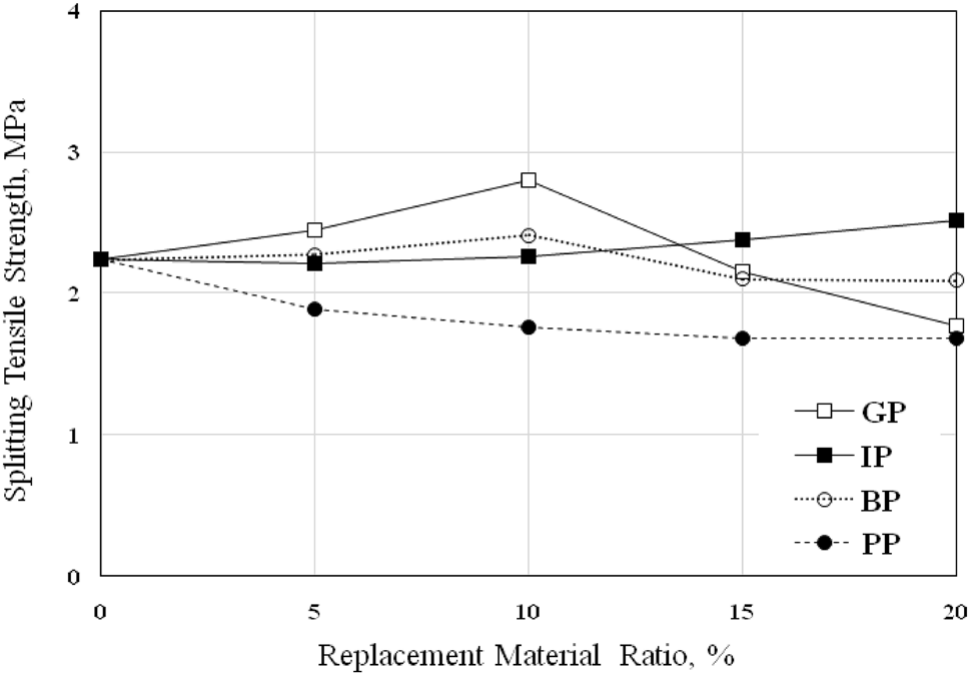
Splitting Tensile Strength of Concrete *f*_*t*_ with various replacement ratios.

The concrete’s splitting tensile strength, *f*_*t*_, was determined using cylinder specimens tested after 28 days; the results are listed in Table 7 and shown in Fig. 8.

**Table 7:** Splitting tensile strength of concrete, *f*_*t*_*.*

MIX	Replacement Ratio (%)	Splitting Tensile Strength, *f*_*t*_ (MPa)				
		0	5	10	15	20
GP		2.24	2.45	2.80	2.15	1.77
IP		2.24	2.21	2.26	2.38	2.52
BP		2.24	2.27	2.41	2.10	2.09
PP		2.24	1.89	1.76	1.68	1.68

The *f*_*t*_ results exhibited the same behavior of compressive strength as shown in Fig. 8; increasing GP and BP contents led to an increase in *f*_*t*_ for mixes GP-5, GP-10, BP-5, and BP-10, which were increased by 9.38%, 25%, 1.34%, and 7.59% respectively. The results showed that the optimum percentage of GP and BP to achieve the maximum enhancement in *f*_*t*_ was 10% compared to control mixes, the same results obtained by^29,31^. After this ratio, increasing GP and BP contents led to a decrease in *f*_*t*_ of concrete; the *f*_*t*_ of mixes GP-15, GP-20, BP-15, and BP-20 decreased by 4%, 21%, 6.3%, and 6.7%, respectively.

Furthermore, the behavior of the *f*_*t*_ of the concrete with IP replacement material was similar to compressive strength except for IP-5, which gives nearly the same strength as the control mix; the *f*_*t*_ of mixes IP-10, IP-15, and IP-20 was increased by 1%, 6.3%, and 12.5% respectively. IP-20 gives a higher rate of enhancement in *f*_*t*_ compared to compressive strength; the same observation was obtained by Ghannam^27^.

On the other hand, the results showed that the *f*_*t*_ of concrete with PP continued to decrease with the increase in PP ratio; the *f*_*t*_ decreased by 15.6%, 21.4%, 25%, and 25% for mixes PP-5, PP-10, PP-15, and PP-20 respectively. The same results were obtained in compressive strength caused by the weakness in the bond between the plastic particles and mortar. The same results were reported in^32,33^.

### Flexural strength

**Figure 9:**
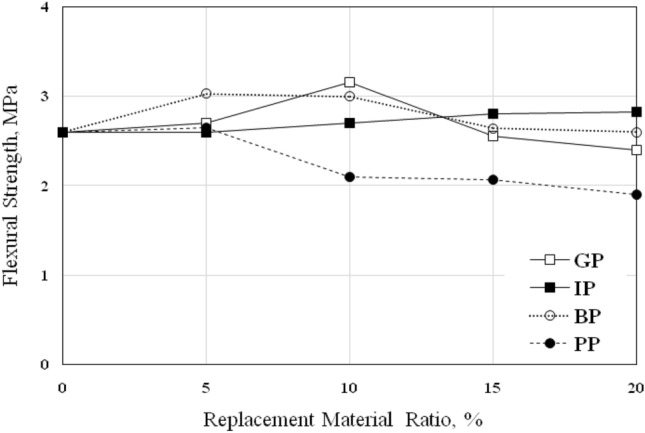
Flexural Strength of Concrete* f*_*f*_ with various replacement ratios.

The flexural strength, *f*_*f,*_ of the concrete was determined using a beam specimen of 10 × 10 × 50 mm. The *f*_*f*_ results are listed in Table 8 and shown in Fig. 9.

**Table 8:** Flexural strength of concrete, *f*_*f*_.

MIX	Replacement Ratio (%)	Flexural Strength, *f*_*f*_ (MPa)				
		0	5	10	15	20
GP		2.6	2.70	3.16	2.55	2.40
IP		2.6	2.60	2.70	2.80	2.82
BP		2.6	3.03	3.00	2.64	2.60
PP		2.6	2.65	2.10	2.07	1.90

As seen in Fig. 9, increasing BP and IP replacement materials increase the *f*_*f*_ of the concrete; in BP mixes, BP-5 and BP-10 give nearly the same enhancement of 16.5% and 15.4%, respectively. In contrast, BP-15 and BP-20 show similar values of the *f*_*f*_ of the control mix. The *f*_*f*_ of IP mixes continued to increase with the increase of IP ratio; the *f*_*f*_ increased to reach 8.5% in the IP-20 mix. The optimum percentage of GP to achieve the maximum increase in *f*_*f*_ was 10% which increased by 21.5%, while the *f*_*f*_ of GP-15 and GP-20 decreased by 2% and 7.6%, respectively.

Figure 9 demonstrates the *f*_*f*_ behavior of PP concrete mixes. It is clear that the *f*_*f*_ exhibited the same behavior of compressive strength and splitting tensile strength except for PP-5, which increased by 2%; the *f*_*f*_ of PP-10, PP-15, and PP-20 decreased by 19.2%, 20.4%, and 27% compared to control mix, this either may be due to weakness bond observed between PP and the concrete which lead to a reduction in the strength values.

### Impact strength

**Figure 10:**
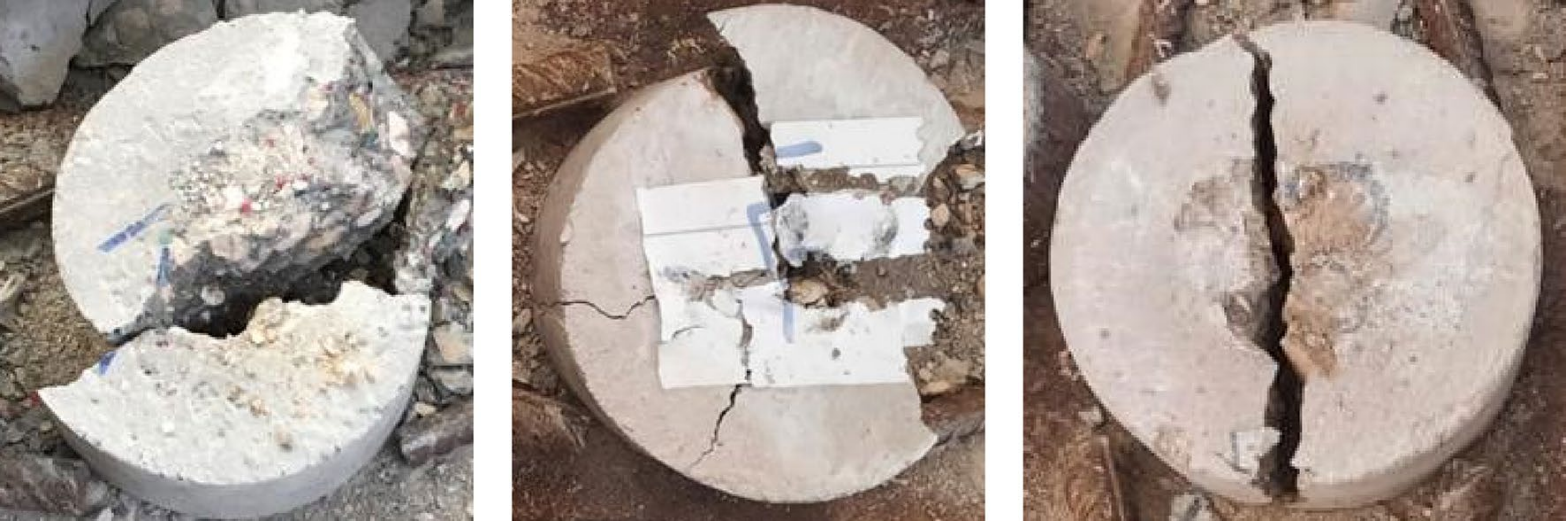
Failure modes of tested specimens.

**Figure 11:**
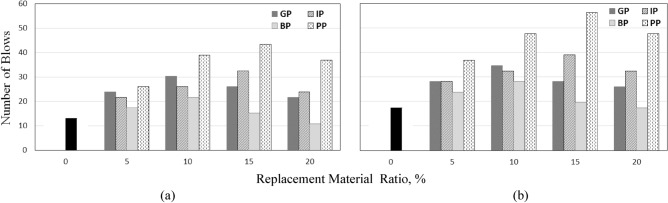
Effect of replacement materials and their ratio on the number of blows at; (**a**) first crack and (**b**) failure.

**Figure 12:**
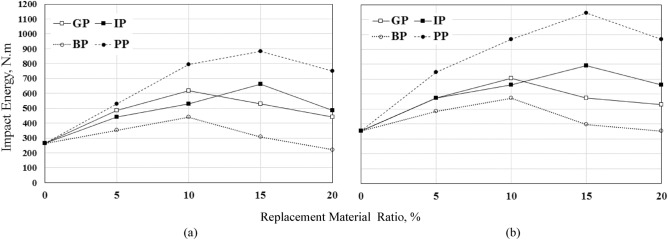
Effect of replacement materials and their ratio on the impact energy, *u*_*dw,*_ at; (**a**) first crack and (**b**) failure.

The impact strength was determined using the drop-weight impact test. The number of blows required for the first crack that occurred at the specimen's top surface was recorded. Also, the number of blows required for the ultimate failure was recorded. The first crack was determined by visual observation, while the ultimate failure is defined in terms of the number of blows required to open the cracks in the specimens enough to enable the collapsed pieces to touch three of the four lugs on the base plate, Fig. 10 shows the failure modes of the tested specimens. The number of blows required for the first crack and failure was recorded, and the draw-weight impact energy (*IE*) was determined and listed in Table 9 and shown in Figs. 11 and 12. The following formula can calculate the impact energy^37–39^:

$$ IE = {\mathbf{Nmgh}}\left( {{\text{N}} \cdot {\text{m}}} \right) $$

where **N** was the number of blows, **m** was the mass of the drop hammer (4.54 kg), **g** was gravity acceleration (9.81 m/sec2), and **h** was the height of the drop hammer (0.457 m).

**Table 9:** Number of blows and impact energy of concrete.

MIX	Number of blows		Impact energy,* IE*_*,*_ (Nm)			
	First crack	Failure	At first crack (*IE*_*i*_)	*IE*_*i*_ enhancement %	At failure (*IE*_*f*_)	*IE*_*f*_ enhancement %
S-0	13	17	264	–	352	–
GP-5	24	28	485	84	573	63
GP-10	30	35	617	134	705	100
GP-15	26	28	529	100	573	63
GP-20	22	26	441	67	529	50
IP-5	22	28	441	67	573	63
IP-10	26	33	529	100	661	88
IP-15	33	39	661	150	793	125
IP-20	24	33	485	84	661	88
BP-5	17	24	352	33	485	38
BP-10	22	28	441	67	573	63
BP-15	15	20	308	17	396	13
BP-20	11	17	220	− 17	352	0
PP-5	26	37	529	100	749	113
PP-10	39	48	793	200	969	175
PP-15	43	56	881	234	1145	225
PP-20	37	48	749	184	969	175

*IE* = Impact energy.

Table 9 and Figs.11 and 12 show the effect of changing the replacement materials type and the replacement ratio on the number of blows and impact energy *IE* on the first crack (*IE*_*i*_) and failure (*IE*_*f*_). It is clear that the number of blows then the impact energy at first crack and failure increased with increasing the GP and BP ratio to 10%. Using 5%, 10%, 15%, and 20% of GP increases the impact energy at first crack by 84%, 134%, 100%, and 67%, respectively, and the impact energy at failure by 63%, 100%, 63%, and 50%, respectively. 10% GP has the best enhancement in impact energy at both the first crack and failure. On the other hand, using BP with ratios 5%, 10%, and 15% increases the impact energy at first crack by 33%, 67%, and 17%, respectively, and the impact energy at failure by 38%, 63%, and 13% respectively. 20% of BP decreases the impact energy at the first crack and is close to the control mix at failure. The same observation is that 10% of BP has the best enhancement in impact energy at both first crack and failure as GP. The *IE* followed the same compressive, splitting tensile, and flexural strength trend. It can be concluded that 10% of GP and BP is the optimum percent that gives the maximum enhancement ratio.

Using 5%, 10%, 15%, and 20% of IP as a partial replacement of sand increases the impact energy at first crack by 67%, 100%, 150%, and 84% and at failure by 63%, 88%, 125%, and 88% compared to the control mix. IP increased the number of blows and *IE* even by 15% replacement percent; it was the optimum ratio; the increase in *IE* for the first crack and failure was 150% and 125%, respectively. Unusually using 20% of IP decreases the numbers of blows and *IE* compared to other ratios. The maximum enhancement in the number of blows and *IE* when using PP with 15%.

Using PP increases the *IE*_*,*_ unlike the other mechanical properties. Noticeably significant improvement in impact energy at both first crack or failure when using PP partially replaces sand. Using 5%, 10%, 15%, and 20% of PP increases the impact energy at first crack by 100%, 200%, 234%, and 184%, respectively, and at failure by 113%, 175%, 225%, and 175%, respectively, compared to the control mix. PP shows positive behavior against impact loading due to the flexibility of the PP. The flexibility was attributed to PP's high ductility and a high potential for absorbing energy, which improves the concrete ductility and leads to absorbing the impact load, as obtained by^32,33,40^. The optimum ratio which gives the maximum enhancement was 15%, but the main disadvantage is the reduction in the compressive strength when increasing the PP replacement ratio. It is highly recommended to be used in construction subjected to impact load.

## Conclusion

The mechanical properties, i.e., compressive strength, *f*_*c,*_ splitting tensile strength, *f*_*t,*_ flexural strength, *f*_*f,*_ and impact energy *IE* of concretes containing GP, IP, BP, and PP as a partial replacement of sand were studied, and the following conclusions can be drawn:

- All wastes except PP succeeded in completely replacing fine aggregate and getting reasonable compressive strength of mortar.
- The IP mortar showed the highest compressive strength, equal to 110.3% of the control mix, due to the IP advantages of IP in strength, surface texture, and shape.
- Using IP as a partial replacement of sand in concrete showed good enhancement in all mechanical properties studied; compressive, splitting tensile, flexural strength, and impact energy compared to the normal concrete.
- The mechanical properties of concrete increased with the increase of GP and BP ratios. The optimum value is obtained at a 10% replacement ratio; after that, increasing the replacement ratio of GP and BP decreases the mechanical properties of concrete.
- The compressive, splitting tensile, flexural strength, and impact energy of GP mixes increased by 11.7%, 25%, 21.5%, and 134% for the first crack and 100% for failure, respectively, when using the optimum ratio of 10% of GP, using the optimum ratio of 10% of BP. Increase the compressive, splitting tensile, flexural strength, and impact energy by 12.9%, 7.5%, 15.4%, and 67% for the first crack and 63% for failure, respectively.
- Although using PP as a partial substitute for sand reduces the mechanical properties of concrete, it significantly affects the enhancement of impact energy. Impact energy increased by 234% for the first crack and 225% for the failure when using 15% PP.
- The current study shows the viability of producing concrete with waste materials. This will motivate producers and environmental organizations to gather and store these wastes. Future studies should also examine the life-cycle costs of using these waste materials.

## Data Availability

All data generated or analyzed during this study are included in this published article.
